# Resistance to apicoplast translational inhibitors in *Plasmodium*

**DOI:** 10.1016/j.ijpddr.2025.100597

**Published:** 2025-05-10

**Authors:** Jessica L. Home, Geoffrey I. McFadden, Christopher D. Goodman

**Affiliations:** School of BioSciences University of Melbourne, VIC, 3010, Australia

**Keywords:** Malaria transmission, Drug resistance, Plastid translation, Doxycycline, Azithromycin, Clindamycin

## Abstract

The spread of drug-resistant *Plasmodium* threatens malaria control efforts. Thus, understanding the mechanisms of resistance is crucial for implementing effective treatments and prevention strategies. The prokaryote-like translational machinery encoded by the apicoplast is the apparent target of several antibiotics with antimalarial activity. Among them, doxycycline and clindamycin are widely used for malaria treatment and/or chemoprophylaxis. However, the mechanisms underlying *Plasmodium* resistance to apicoplast-targeting antibiotics, and the evolution of such resistance mechanisms, remain largely unknown. In this review, we summarise reported cases of resistance to apicoplast translational inhibitors uncovered in either laboratory or clinical settings. We highlight the potential evolutionary pathway of doxycycline resistance, explore why resistance to these antibiotics remains rare in the field, and assess whether expanding their use in malaria treatment and prevention is a viable strategy.

## Highlights

• Mutations in apicoplast translational machinery confer *Plasmodium* resistance to azithromycin and clindamycin.
• No reports of *Plasmodium* resistance to doxycycline.
• Doxycycline resistance may evolve through a stepwise process.
• Parasite fitness and multi-target drugs influence the evolution of resistance.
• Potential in using antibiotics more widely for malaria treatment and prevention.

## Graphical abstract

**Figure:**
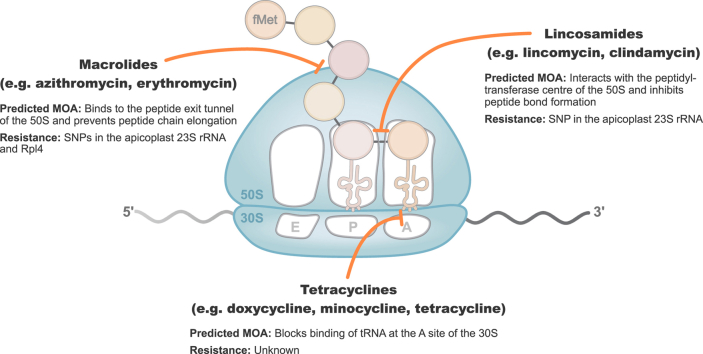

## 1 Resistance to apicoplast protein synthesis inhibitors in malaria parasites

Drug resistance poses a significant challenge in malaria control, with resistance emerging against almost all clinically implemented antimalarials (White, 2004). This continuous evolution of resistance necessitates the search for new drug targets and compounds to replace those that have become ineffective. Given that resistance is an inevitable consequence of antimalarial use, preserving older therapies remains crucial, particularly as antimalarials are our only option for treating infected individuals.

Several classes of antibiotics exhibit antimalarial activity and play an integral role in controlling malaria. For instance, doxycycline is regularly prescribed as a chemoprophylaxis for travellers to malaria-endemic regions (Tan and Abanyie, 2023; World Health Organization, 2012), and tetracycline, doxycycline or clindamycin in combination with artesunate or quinine are used for cases where artemisinin combination therapies (ACTs) fail, although this second-line treatment regime is generally considered unfavourable (World Health Organization, 2024). While antibiotics are well characterised and generally well-tolerated, their delayed onset of action against *Plasmodium*, and concerns regarding resistance, has limited their widespread use in malaria treatment.

The apicoplast, an essential relict plastid of malaria parasites derived from a photosynthetic bacterial endosymbiont (McFadden, 2011), is the proven target of antimalarial antibiotics (Dahl and Rosenthal, 2007; Uddin et al., 2017). These antibiotics apparently act on targets encoded either by the apicoplast genome or by nuclear genes encoding proteins trafficked to the apicoplast (Dahl and Rosenthal, 2008). To preserve these valuable therapies, an understanding of antibiotic resistance mechanisms and their evolution in *Plasmodium* is essential. This section explores the mechanisms of resistance to apicoplast translational inhibitors, as identified in both laboratory and clinical settings, and considers the similarities between antibiotic resistance in malaria parasites and bacteria.

### 1.1 Macrolides

The macrolide class comprises numerous broad-spectrum antibiotics that contain a macrocyclic lactone ring (Dinos, 2017). Early studies of macrolide efficacy against *Plasmodium* focused on the progenitor macrolide erythromycin, but more recent derivatives, such as azithromycin and clarithromycin, exhibit greater potency and tolerability (Du Plessis et al., 2012; Uddin et al., 2017), with azithromycin being the most extensively studied. Azithromycin likely inhibits apicoplast protein translation by binding to the apicoplast 50 S large ribosomal subunit at the exit tunnel, where it is proposed to interact with the 23 S rRNA and ribosomal protein L4 (Rpl4) to block transpeptidation (Sidhu et al., 2007). *In vitro* studies have identified multiple single polymorphisms (SNPs) in both the apicoplast 23 S rRNA and *rpl4* genes that confer resistance in *P. falciparum* and *P. berghei* parasites (Buchanan et al., 2024; Goodman et al., 2013; Sidhu et al., 2007; Wilson et al., 2015). Notably, no single mutation is consistently associated with azithromycin resistance, as each study reports a distinct mutation. This variability may result from differences in selection protocols or species/strain-specific genetic backgrounds, or simply that many paths to resistance exist for azithromycin.

Although mutations in either 23 S rRNA or *rpl4* genes confer high levels of azithromycin resistance alone, the existence of multiple resistance-conferring mutations raises the possibility that parasites harbouring mutations in both genes could exhibit enhanced resistance. Independent selections for azithromycin-resistant *P. falciparum* 7G8 and Dd2 strains revealed an identical point mutation (G76V) in *Pfrpl4* (Sidhu et al., 2007). In addition, a SNP in domain I of the 23 S rRNA was detected in azithromycin-resistant 7G8 parasites, though it did not appear to confer any significant additional resistance to the antibiotic (Sidhu et al., 2007). This may be due to its distance from the primary binding site of azithromycin in domains II, IV and V of the 23 S rRNA. It is not yet clear if the mutation in the 23 S rRNA of 7G8 parasites arose by chance or if it serves as a compensatory mutation to maintain parasite fitness. Further investigation is required to determine whether resistance conferring SNPs in both the 23 S rRNA and *Pfrpl4* can co-exist and act synergistically to enhance resistance.

### 1.2 Lincosamides

The lincosamide class encompasses a relatively small group of compounds, with clindamycin being the most potent and widely used against *Plasmodium* (Spížek and Řezanka, 2017). Until recently, quinine-clindamycin was the preferred treatment for uncomplicated *P. falciparum* malaria in pregnant women during their first trimester. However, re-evaluation of the safety of ACT treatment during first trimester pregnancy revealed artemether-lumefantrine as a more tolerable and efficacious treatment option, and it is now the recommended treatment option (World Health Organization, 2024).

In bacteria, clindamycin inhibits protein elongation by blocking the peptidyl transferase reaction on the large ribosomal subunit (Spížek and Řezanka, 2004). To date, there is only one report of *Plasmodium* resistance to clindamycin in the field. Whole genome sequencing of 14 *P. falciparum* patient isolates in the Peruvian Amazon identified two point mutations in the apicoplast-encoded 23 S rRNA. One mutation, A1875C, confers clindamycin resistance, but the second identified mutation, A2409U, did not confer resistance *in vitro* (Dharia et al., 2010). The A1875C mutation is in the peptidyl transferase cavity—the active site of peptide bond formation and the predicted binding site of clindamycin (Dharia et al., 2010; Douthwaite, 1992). The peptidyl transferase centre is highly conserved across all domains of Life (Doris et al., 2015), and modification at the corresponding A1875 site in bacteria and algal chloroplasts confers resistance to clindamycin (Dharia et al., 2010; Harris et al., 1989; Poehlsgaard et al., 2005; Vester and Douthwaite, 2001).

Whether or not these apicoplast 23 S rRNA SNPs arose due to clindamycin selection pressure is unclear. Frequent use of clindamycin for treatment of bacterial infections may have led to the inadvertent selection of clindamycin resistant *Plasmodium.* In addition, historical use of quinine combinations with clindamycin or tetracycline as third-line therapies in Peru during the 1990s (Griffing et al., 2013), and the prolonged use of quinine-clindamycin for treating malaria in first-trimester pregnant women, may have also contributed to the clindamycin selection pressure. Furthermore, Peru is considered a low-endemic malaria region, with most cases caused by *P. vivax* (Hogan et al., 2024). In general, genetic recombination occurs less frequently in such low-transmission settings, reducing the effects of selection and thus increasing the likelihood of mutational drift (Escalante et al., 2022). However, since the apicoplast follows a uniparental mode of inheritance (Collier et al., 2025; Creasey et al., 1994), genetic recombination will not occur during the parasite's sexual stage and therefore selection will affect the frequency of the 23 S rRNA mutation to a larger extent. In sum, there is insufficient data to decide if clindamycin resistance is readily selected for and transmissible in *Plasmodium*. Nevertheless, it would seem prudent to survey for mutations in the active site of the apicoplast 23 S rRNA where clindamycin is deployed.

Clindamycin resistance has been generated experimentally in *P. berghei*. Continuous *in vivo* exposure to increasing clindamycin concentrations resulted in high-level, stable resistance following 42 successive passages over a 300 days (Jacobs and Koontz, 1976). Despite the slow emergence of resistance, no blood-stage growth defect was observed (Jacobs and Koontz, 1976). While the underlying mechanism was not determined, a subsequent study confirmed that resistance was not due to decreased drug uptake (Koontz et al., 1979). Notably, an attempt to generate clindamycin resistance in *P. falciparum* through prolonged drug exposure *in vitro* failed to generate resistant parasites (Seaberg et al., 1984). These findings suggest that clindamycin resistance is not easily acquired, though advances in *Plasmodium* culturing and drug selection methodologies should provide further insights into the evolution of clindamycin resistance.

### 1.3 Tetracyclines

Tetracycline, doxycycline and minocycline are long-acting antibiotics belonging to the tetracycline class (Gaillard et al., 2015). Evidence of *Plasmodium* resistance to tetracyclines remains sparce, despite use of doxycycline as a chemoprophylaxis (Tan and Abanyie, 2023; World Health Organization, 2012), and extensive treatment against non-malarial pathogens with doxycycline. The only reported case of tetracycline resistance in *Plasmodium* involved minocycline resistance in rodent malaria *P. berghei*, although this resistance was unstable. After 600 days of continuous *in vivo* exposure, parasites exhibited a six-fold increase in resistance to minocycline*,* but susceptibility reverted to wild type levels following drug-free passages (Jacobs and Koontz, 1976). The difficulty in generating high-level, stable resistance aligns with the lack of *Plasmodium* doxycycline resistance observed in the field. This unusual scenario for malaria parasite drug resistance is discussed in detail below.

In prokaryotes, tetracyclines target the small ribosomal subunit and inhibit translation by blocking binding of incoming charged tRNAs (Roberts, 1996). Compared to clindamycin and azithromycin, there is limited evidence to suggest that tetracyclines target apicoplast translation in *Plasmodium* as no associated resistance mutations have been identified. Thus, *in vitro* selection of tetracycline resistance, or proteomics studies to assess translation of apicoplast-encoded peptides during tetracycline treatment, might provide insight.

### 1.4 Cross-resistance

Cross-resistance among bacterial translational inhibitors is well-documented in prokaryotes (Spížek and Řezanka, 2004). However, studies investigating antibiotic resistance in *Plasmodium* have not reported cross-resistance between different antibiotic classes or with other antimalarials. Intriguingly, azithromycin-resistant *P. falciparum* 7G8 parasites harbouring the point mutation in the 23 S rRNA exhibited a two-fold increase in sensitivity to tetracycline, doxycycline, and thiostrepton, but an azithromycin-resistant Dd2 strain that lacked 23 S rRNA SNPs did not exhibit cross sensitivity (Sidhu et al., 2007). This hypersensitivity was specific to apicoplast translational inhibitors, as chloroquine susceptibility remained unchanged (Sidhu et al., 2007). Similarly, clindamycin resistance in the related parasite *Toxoplasma gondii* harbouring a mutation in the apicoplast 23 S rRNA displayed a slight hypersensitivity to doxycycline but reduced sensitivity to azithromycin and chloramphenicol (Camps et al., 2002). Although these data are preliminary, they proffer an attractive scenario where combination antibiotic therapies might mitigate resistance development because resistance-conferring 23 S rRNA mutants for one drug cause cognate hypersensitivity to other apicoplast inhibitors. Further studies into cross resistance could be worthwhile.

### 1.5 Are bacterial antibiotic resistance mechanisms mimicked in *Plasmodium*?

Since the discovery of the antimalarial activity of antibiotics, and the identification of a 35 kb prokaryote-like genome of the apicoplast, it has been widely presumed that the targets of antibiotics reside in the endosymbiotic organelle. Concomitantly, it was anticipated that malaria parasite resistance mechanisms to prokaryotic translational inhibitors would mimic what is observed in bacteria. However, the unique evolutionary history of the apicoplast has resulted in distinct differences in ribosome structure and translational mechanisms. For example, the apicoplast ribosome apparently lacks a 5 S rRNA (Wilson et al., 1996), as well as the essential (in bacteria) 5 S rRNA-binding protein Rpl5 and several other small subunit ribosomal proteins conserved in bacteria (Gupta et al., 2014). These differences likely alter the structure and function of the apicoplast ribosome, but how significant these differences are to antibiotic activity awaits the solving of the structure of the apicoplast ribosome. *Plasmodium* also appears to lack erythromycin ribosome methylase (*erm*) genes, which in bacteria confer resistance to macrolide, lincosamide and streptogramin B (MLS_B_) antibiotics (Douthwaite et al., 2004; Spížek and Řezanka, 2017; Vester and Long, 2013). Nevertheless, resistance mechanisms for clindamycin and azithromycin in *Plasmodium* align well with bacterial resistance mechanisms. A key limitation to dissecting apicoplast drug resistance is the ongoing lack of ability to perform genetic modification of the apicoplast genome.

**Fig. 1:**
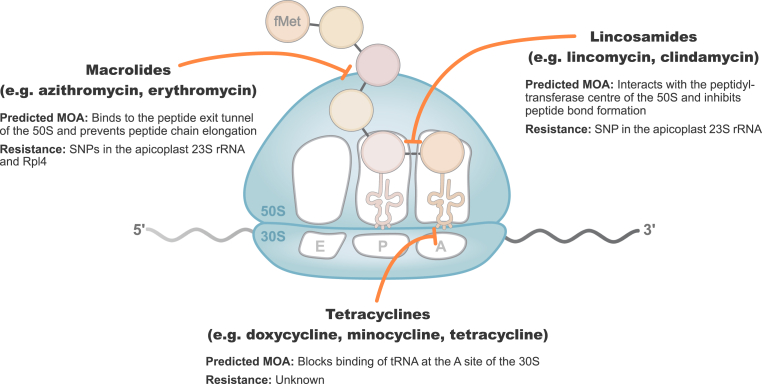
Graphical illustration of apicoplast protein translation in *Plasmodium*. Predicted mechanisms of action (MOA), and known resistance mechanisms of antibiotics within the macrolide, lincosamide and tetracycline classes are depicted.

The translational process of the apicoplast is not yet well understood. For instance, in bacteria and endosymbiotic organelles such as plastids and mitochondria, translation canonically begins with a formylated initiator methionine tRNA (fMet-tRNA^fMet^) (Fig. 1) (Habib et al., 2016; Laursen et al., 2005). The N-terminal formyl group is rapidly removed by peptide deformylase (PDF), an essential enzyme for downstream protein processing, function and stability (Bingel-Erlenmeyer et al., 2008; Giglione et al., 2004). Apicoplast translation is predicted to follow this mechanism, as genes encoding key enzymes such as methionyl-tRNA formyltransferase (FMT) and PDF are present in *Plasmodium,* and are either predicted or confirmed to localise to the apicoplast (Habib et al., 2016; Meinnel, 2000; Pütz et al., 2010; Tonkin et al., 2004). To date, however, none of these pathways have been confirmed biochemically. This leaves us open to misinterpreting the mode of action of antimalarial antibiotics, actinonin being the case-in-point.

Actinonin, a naturally occurring antibiotic that targets PDF in bacterial systems, has antimalarial activity and inhibits recombinantly expressed *Pf*PDF (Bracchi-Ricard et al., 2001). This data led to the assumption that apicoplast PDF is the target of actinonin. However, unlike other apicoplast translation inhibitors, actinonin exhibits fast parasite-killing activity rather than the expected delayed death drug response (Uddin et al., 2017). Eventually, resistance studies in *T. gondii* and *P. falciparum* identified the putative metalloprotease FtsH1, rather than apicoplast PDF, as the primary target of actinonin (Amberg-Johnson et al., 2017; Goodman et al., 2020), Actinonin thus serves as a cautionary tale about assuming the target of an antibacterial will be the orthologue in the apicoplast. What now needs to be determined is whether apicoplast PDF is indeed a valid drug target. Fortunately, a range of actinonin analogues are available, and testing these against *Plasmodium* could be fruitful. Also, PDF is nucleus-encoded and the product targeted to the apicoplast, so reverse genetic approaches to confirm essentiality are also worth pursuing.

## 2 Is doxycycline resistance on the horizon?

*Plasmodium* resistance is a major challenge in controlling and eradicating malaria. Malaria parasites have an extraordinary ability to adapt to environmental pressures, as evidenced by their resistance to nearly every antimalarial deployed in clinical settings. Doxycycline has a role in the prevention and treatment of malaria however, the frequent use of doxycycline and other antibiotics in the treatment of bacterial and non-malarial diseases significantly contributes to the antibiotic selection pressure, which might result in so-called bystander resistance. Yet, despite widespread use of doxycycline, no high-level *Plasmodium* resistance to the antibiotic has been reported. How has doxycycline escaped the seemingly inevitable emergence of resistance? In this section, we explore how *Plasmodium* parasites may be evolving in response to doxycycline pressure. The mechanisms that may limit the emergence of doxycycline resistance are considered later in this review.

Doxycycline is primarily prescribed as a chemoprophylaxis but is also occasionally used in combination with other antimalarials for treatment of malaria (World Health Organization, 2024). Before its FDA approval for prophylactic use in 1994 (Gaillard et al., 2015; Tan et al., 2011), large-scale efficacy studies were most commonly conducted in military personnel deployed to malaria-endemic regions (Brisson and Brisson, 2012; National Academies of Sciences, 2020; Sanchez et al., 1993; Shanks et al., 1995). Generally, doxycycline is regarded as a highly effective and well-tolerated chemoprophylactic antimalarial (Tan et al., 2011), though occasional failures have been reported. Whilst most cases of prophylactic breakthrough are attributed to insufficient dosing or non-adherence to the prescribed regime (Brisson and Brisson, 2012; Pang et al., 1988), the possibility of *Plasmodium* resistance to doxycycline cannot be ruled out. Notably, a case study of a French soldier deployed to the Central African Republic documented infection by *P. falciparum* despite serum doxycycline concentrations within the expected range, suggesting proper adherence to prophylaxis (Madamet et al., 2015). Although this incident raises the possibility of emerging resistance, susceptibility of this parasite to doxycycline was unfortunately not assayed by *in vitro* testing.

### 2.1 Is *Plasmodium* evolving to doxycycline pressure?

The evolution of drug resistance is complex, being influenced by multiple factors that determine the likelihood of resistance mutations arising and spreading (White, 2004). In many cases, resistance evolves in a step-wise manner, whereby initial or precursor mutations create a genetic foundation upon which high-level resistance mutations can later accumulate (Mita et al., 2014). Resistance to most antimalarials is considered to arise in such a step-wise manner (Klein, 2013). For example, resistance to antifolates arose through accumulation of multiple mutations that ultimately resulted in high levels of resistance (Heinberg and Kirkman, 2015; Plowe et al., 1997). Generally, these precursor mutations directly contribute to reduced drug susceptibility but may also mitigate the fitness costs associated with resistance-conferring mutations (Heinberg and Kirkman, 2015; Nair et al., 2008; Travassos and Laufer, 2009). Identifying and understanding the development of these genetic markers is crucial for resistance surveillance to avoid development of full-blown resistance.

Studies of *P. falciparum* isolates with reduced doxycycline sensitivity provide insight into the potential evolution of doxycycline resistance in malaria. In French Guiana, quinine-doxycycline combination therapy was introduced for treatment of uncomplicated *P. falciparum* malaria in 1995 and remained a first-line therapy until its replacement by artemether-lumefantrine in 2002 (Legrand et al., 2008; Mura et al., 2015). During this period, an increase in the mean doxycycline IC50 was observed among *P. falciparum* isolates from French Guiana, rising from 9.6 μM in 1996–1999 to 13.1 μM in 2005. This rise in doxycycline tolerance coincided with increased doxycycline use (Legrand et al., 2008; Tan et al., 2011). A subsequent analysis of patient isolates across Africa revealed phenotypic variations in doxycycline susceptibility, with 1.2 % of 747 tested samples exhibiting high IC50 values (>35 μM) (Briolant et al., 2009). The mean doxycycline IC50 values reported in these studies is dramatically higher than those observed in culture-adapted laboratory strains (Uddin et al., 2017). However, inconsistencies in the duration of *in vitro* assays may account for some of these discrepancies, as IC50 values in field isolates were determined using growth assays varying in length from 42 to 48–72 h (Achieng et al., 2014; Briolant et al., 2009; Legrand et al., 2008; Tan et al., 2011). Given that doxycycline is a classic second-cycle killer or delayed death drug (Dahl and Rosenthal, 2007), a minimum of a 96-h drug assay is required for accurate and comparable IC50 estimates (Dahl and Rosenthal, 2007; Uddin et al., 2017).

To identify genetic markers associated with the increase in doxycycline tolerance in these parasites, sequencing analysis was performed on genes implicated in doxycycline resistance in bacteria (Briolant et al., 2010). Two genes, *pfmdt* (PF3D7_0516500) and *pftetQ* (PF3D7_1235400), had increased copy numbers in parasites exhibiting high IC50 values, alongside a decrease in the KYNNNN repeat motif in *pftetQ* (Briolant et al., 2010; Madamet et al., 2015). *Pfmdt* and *pftetQ* share sequence similarities with tetracycline resistance-conferring genes in bacteria, where they encode membrane-associated efflux proteins and ribosomal protection proteins respectively (Achieng et al., 2014; Briolant et al., 2010; Connell et al., 2003; Grossman, 2016; Thaker et al., 2009). Interestingly, sequencing of the isolate from the aforementioned prophylactic failure case revealed amplification of *pfmdt* and *pftetQ* genes and a decrease in the KYNNNN motif, further implicating these markers in doxycycline tolerance (Madamet et al., 2015).

However, conflicting data complicates the interpretation of these findings. Although Achieng et al. (2014) linked the KYNNNN motif reduction to doxycycline tolerance, gene amplification of *pfmdt* and *pftetQ* was not consistently present in highly tolerant isolates (>35 μM IC50) (Achieng et al., 2014). Other studies also failed to establish a significant correlation between copy number variants in *pfmdt* and *pftetQ* and doxycycline tolerance (Gaillard et al., 2012; Mura et al., 2015). Nonetheless, a later study by Gaillard et al. (2013) suggested that the initial lack of statistical significance may have been due to a low IC50 cutoff (>25 μM) used to define highly tolerant IC50 isolates. Using updated resistance criteria, a later study found a significant association between *pfmdt* and *pftetQ* and doxycycline tolerance (Gaillard et al., 2013). The fact that these markers are not universally present in all highly doxycycline tolerant isolates indicates that additional resistance-conferring mutations may be at play. For instance, mutations in the ribosomal proteins and the prokaryotic 16 S rRNA confer tetracycline resistance in bacteria (Grossman, 2016; Lupien et al., 2015; Ross et al., 1998). Unfortunately, many studies investigating doxycycline resistance in *P. falciparum* isolates did not include analysis of all implicated ribosomal proteins and/or the apicoplast 16 S rRNA.

Ultimately, functional validation studies are required to confirm whether *pfmdt* and *pftetQ* amplifications directly contribute to increased doxycycline tolerance. For example, the addition of tuneable promoters via genetic modification to drive overexpression of *pfmdt* and *pftetQ* in parasites *in vitro* should be useful in assessing any relationship between doxycycline tolerance and increased gene copy numbers. Additional analyses of candidate resistance genes are also critical to elucidate the molecular basis of doxycycline resistance in *P. falciparum*. A deeper understanding of these mechanisms is essential for monitoring the evolution of resistance and ensuring the continued efficacy of doxycycline as a malaria prophylactic and treatment option.

## 3 Why so little antibiotic resistance in *Plasmodium*?

Antibiotics are widely used for prevention and treatment of malaria and a range of other infections. Given their extensive use, it is surprising that resistance against apicoplast inhibitors in *Plasmodium* is not more commonly observed—especially considering the rapid selection and spread of resistance to frontline antimalarials. One simple explanation is that antibiotics are not typically administered to highly infected individuals, so the number of parasites undergoing resistance selection is perhaps less than for frontline drugs.

Antibiotics are widely used in the prevention of malaria and non-malarial diseases due to their tolerability and cost-effectiveness. For instance, doxycycline is commonly prescribed to travellers to malaria-endemic regions, while azithromycin is frequently used for mass drug administration (MDA) programs directed against various neglected tropical diseases (World Health Organization, 2020). It is thus likely that frequent use of these antibiotics with antimalarial activity against non-malarial diseases creates a selective pressure that could inadvertently select for resistance in the malaria parasite. Interestingly, a study examining *P. falciparum* isolates from villages where azithromycin MDA was implemented for trachoma prevention over a decade revealed no plausible resistance-conferring SNPs in *Pfrpl4*, a gene frequently associated with azithromycin resistance (Schachterle et al., 2014). The apparent lack of *Plasmodium* resistance to widely used apicoplast translational inhibitors, particularly doxycycline and azithromycin, is thus puzzling. We discuss two hypotheses to explain this phenomenon.

First, resistance mechanisms against doxycycline and azithromycin could impose a significant fitness cost on the parasite, reducing likelihood of transmission of resistance mechanisms. Second, the antimalarial activity of these antibiotics could involve multiple targets, thus requiring the (mathematically less likely) simultaneous emergence of multiple interdependent mutations for resistance to develop. These two hypotheses are not mutually exclusive, with multiple targets and high fitness costs perhaps combining to further suppress the chance of resistance developing.

### 3.1 Poor fitness

Antimalarial resistance is selected for during the asexual blood stages where parasites are under drug pressure. However, resistance-conferring mutations often come at a cost to parasite fitness, potentially reducing their transmission if they transition into drug-free environments (Rosenthal, 2013). For instance, the decline of chloroquine resistant parasites following the withdrawal of chloroquine in Malawi elegantly illustrates the balance of drug pressure and parasite fitness, with rapid reversion to sensitive genotype following drug withdrawal (Laufer et al., 2006). Indeed, we now understand that fitness costs of drug resistance mutations can be substantial, likely placing severe constraints on the spread of resistance. For instance, we and others found a block in transmission of parasites resistant to the mitochondrial respiratory chain inhibitor atovaquone (Balta et al., 2023; Goodman et al., 2016, 2017). Although atovaquone-resistant parasites are rapidly selected for, they are trapped within the vertebrate host, unable to successfully infect mosquitos and spread from mouse-to-mouse in the case of *P. berghei* or person-to-person in the case of *P. falciparum* (Balta et al., 2023; Goodman et al., 2016, 2017). This so-called ‘genetic trap’ concept also appears to apply to other drugs. A recent study by Buchanan et al. (2024) demonstrated that transmission of azithromycin resistant, Rpl4 mutants in *P. falciparum* and *P. berghei* was inhibited to some degree. Since the apicoplast is more metabolically active during the transmission stages of the life cycle (Buchanan et al., 2022), mutations conferring azithromycin resistance—selected in the blood stages—appear to compromise apicoplast function and reduce parasite fitness in the later, drug-free stages of the life cycle (Buchanan et al., 2024). However, whether the fitness of laboratory-generated resistant mutants translates to the field remains unknown and is challenging to assess. While the generation of clinically relevant atovaquone-resistant parasites validated that such mutants are effectively blocked in transmission (Balta et al., 2023), azithromycin-resistant mutants have yet to be characterised in the field.

Furthermore, Buchanan et al. (2024) identified disparities in the fitness of mutant *rpl4* genes in *P. falciparum* and *P. berghei*. Thus, while *P. falciparum rpl4* mutants appear to develop normally in the mosquito, *P. berghei* azithromycin mutant parasites exhibited severe defects in mosquito-stage development. However, both species showed impaired replication during the liver stage infection *in vivo* (Buchanan et al., 2024). Rodent malaria models are invaluable for studying *in vivo* parasite development, host immunity, and to replicate transmission in a laboratory setting (De Niz and Heussler, 2018). However, the stark differences observed in the transmission of azithromycin resistant *P. falciparum* and *P. berghei* azithromycin mutants (Buchanan et al., 2024) highlights the limitations of relying solely on rodent models to study transmission and drug resistance. These differences likely stem from species-specific metabolic requirements throughout the life cycle, as reviewed in Buchanan et al. (2022).

Whether resistance mutations to clindamycin and doxycycline impose similar fitness costs on parasite transmission remains unknown. However, such fitness deficits may help explain the absence of resistance to apicoplast translational inhibitors in the field.

### 3.2 Doxycycline and azithromycin have multiple targets in *plasmodium*

Combination-based therapies, whereby antimalarials with different targets are paired for prevention and/or treatment of malaria, have significantly reduced the emergence and spread of resistant malaria, likely because mutations conferring resistance to both compounds are less likely to arise contemporaneously (Laxminarayan, 2004; Peters, 1990; White, 1998). Similarly, resistance is less likely to develop against lone antimalarials that hit more than one target compared to those with a single target (Mackinnon and Hastings, 1998). Both doxycycline and azithromycin are proposed to have dual targets in *Plasmodium* (Burns et al., 2020; Dahl et al., 2006; Goodman et al., 2013; Okada et al., 2020; Wilson et al., 2015), which may contribute to the rarity of resistance to these antibiotics.

Recent evidence suggests that doxycycline targets both the apicoplast and mitochondrion in *Plasmodium*. At concentrations below 5 μM, doxycycline disrupts the apicoplast, resulting in delayed, second-cycle antimalarial activity (Dahl et al., 2006; Uddin et al., 2017; Yeh and DeRisi, 2011). However, at higher doxycycline concentrations (>10 μM) parasites are killed in the first erythrocytic cycle (Dahl et al., 2006; Okada et al., 2020), which suggests the existence of a second, faster-acting mechanism of action. The mitochondrion is the suspected ‘fast’ target, as supported by three lines of evidence: (1) tetracyclines reduce mitochondrial function by decreasing DHODH activity (Prapunwattana et al., 1988); (2) biphasic inhibition curves are observed following tetracycline treatment, suggesting the presence of two independent targets (Budimulja et al., 1997); and (3) mitochondrion-encoded gene expression decreases during the first erythrocytic cycle with tetracycline or minocycline treatment (Lin et al., 2002). A recent study proposed an additional, fast-killing activity of doxycycline related to a metal-dependent mechanism within the apicoplast. Exogenous iron rescues *P. falciparum* parasites from doxycycline inhibition at 10 μM but not higher concentrations (Okada et al., 2020). A third doxycycline activity is an intriguing possibility that awaits identification of a specific target and definitive investigation of the interplay between doxycycline's direct effects and its propensity to chelate iron, which can lead to altered drug activity (Faure et al., 2021).

Similar fast-killing activity has also been observed with azithromycin (Burns et al., 2020; Goodman et al., 2013; Wilson et al., 2015). At concentrations above 10 μM, azithromycin inhibits parasite red blood cell invasion, a mechanism of action distinct from its the apicoplast-targeting effects (Burns et al., 2020; Wilson et al., 2015). Attempts to generate resistance to this fast-killing mechanism using azithromycin resistant *Pfrpl4* mutants were unsuccessful (Burns et al., 2020), raising questions about whether both resistance mechanisms can coexist in a single parasite. If resistance mutations act additively, resistance is likely to emerge more rapidly than if both mutations are required to confer azithromycin resistance (Mackinnon and Hastings, 1998). It is thus possible that resistance to higher concentrations of doxycycline and azithromycin requires parasites to acquire both mechanisms of resistance simultaneously, which would significantly reduce the probability of resistance emerging in the field. However, it is unclear if azithromycin or doxycycline plasma concentrations reach high enough *in vivo* to target these fast-acting, secondary mechanisms of action, thus potentially negating the clinical relevance of the second target (Dahl et al., 2006; Newton et al., 2005; Okada et al., 2020).

## 4 Should apicoplast translational inhibitors be used more widely?

### 4.1 Chemoprevention

Preventative chemotherapies are essential in protecting vulnerable groups and non-immune travellers against malaria. Sulfadoxine-pyrimethamine (SP) is predominately used for intermittent preventative treatment of malaria in pregnancy (IPTp) and perennial malaria chemoprevention (PMC) in young children, whilst SP plus amodiaquine is used for seasonal malaria chemoprevention (SMC) as recommended by the WHO (World Health Organization, 2024). However, increasing SP resistance is threatening the efficacy of these chemo preventative strategies (Eijk et al., 2019; Masserey et al., 2025). Clindamycin and azithromycin are cost-effective, extremely well-tolerated and safe in young children and pregnant women, making them potentially attractive options for chemoprevention of malaria (Lell and Kremsner, 2002; Rosenthal, 2016). Mass distribution of azithromycin is regularly used for trachoma prevention in children, however the impact of these programs on malaria prevalence is unclear. Whilst some studies demonstrated significant reductions in the prevalence of malaria following administration of the antibiotic in Africa (Gaynor et al., 2014; Schachterle et al., 2014), others show no significant effect (O'Brien et al., 2017; Oldenburg et al., 2018). Furthermore, there was no reduction in the incidence of malaria when azithromycin was combined with regular seasonal malaria chemoprevention, but the burden of non-malarial illnesses was significantly lower in the group receiving azithromycin (Chandramohan et al., 2019). Overall, the evidence for the effectiveness of azithromycin in malaria prevention is mixed, and further exploration is required to determine whether the antibiotic could be a useful addition in areas with high circulating resistance to chemoprophylaxis antimalarials. Timing azithromycin MDA for diseases such as trachoma with seasonal malaria chemoprevention could be an effective strategy for reducing the burden of malaria and nonmalarial diseases in young children (Gao et al., 2014). If azithromycin resistance is indeed impeded from spreading (Buchanan et al., 2024), then there could be less of a concern about generating an azithromycin resistance problem in malaria parasites.

Many of these antibiotics also exhibit multi-stage inhibition. For instance, tetracyclines are effective in inhibiting hepatic stages of the malaria life cycle (Kumar and Dutta, 1987, 1989; Marussig et al., 1993), whilst azithromycin is effective in targeting both mosquito (Gendrin et al., 2016; Shimizu et al., 2010) and liver (Friesen et al., 2010) stage parasites. In addition, azithromycin accumulates in the liver (Friesen et al., 2010), further highlighting its potential efficacy for use in malaria preventative therapy. Transmission-blocking antimalarials are seen as a useful tool in combination therapies to help prevent the spread of resistance to asexual blood stage inhibitors (Birkholtz et al., 2022), especially as parasites in the blood of a human host far outnumber parasites in the mosquito and liver stages (Sinden, 2001; White, 2004; White and Pongtavornpinyo, 2003), mathematically reducing the chance of resistance arising. Thus, targeting parasites during the population bottleneck stages of the life cycle should be a superior strategy to limit the spread of malaria.

### 4.2 Treatment

For many decades apicoplast translational inhibitors have played an important role in treatment of malaria. The WHO recommends tetracycline, doxycycline or clindamycin in combination-based therapies as a backup for frontline antimalarials and other specific cases (see above) (World Health Organization, 2024). Although apicoplast translation inhibitors must be used in combination due to their slow onset of action, the lack of resistance currently in the field, and the potential fitness costs associated with resistance-conferring mutations, could negate the concerns regarding emerging resistance. There is also a strong argument for the addition of these antibiotics in combination-based therapies in reducing the prevalence of other bacterial infections that are typically concomitant in patients with severe malaria. Additionally, symptoms of other bacterial infections and parasitic diseases are often mistaken as severe malaria, thus the addition of broad-spectrum antibiotics in combination therapies could help account for these misdiagnoses (Noedl, 2009). However, the inclusion of antibiotics in such therapies may result in the selection of bystander resistance and other negative consequences associated with antibiotic intake, such as microbiome dysbiosis, cannot be discounted. While a deeper understanding of how these antibiotics act in combination with artemisinin-based derivatives is required, exploring these drugs in double combination or triple combination-based therapies may be beneficial.

## 5 Concluding remarks

The apicoplast is essential across all life cycle stages of the malaria parasite, making it an attractive drug target. Antibiotics that inhibit apicoplast function are well-characterised and generally safe, and therefore development and implementation of antibiotic-containing antimalarial combinations could be straightforward. However, the use of broad-spectrum antibiotics in malaria prophylaxis and/or treatment raises concerns about bystander resistance in non-target pathogens. Continuous resistance monitoring is therefore crucial to ensure the benefits of their antimalarial application outweigh potential risks.

*Plasmodium* resistance to apicoplast inhibitors appears refractory, particularly in the case of doxycycline. The underlying reasons for this remain unclear, highlighting the need for further investigation into the molecular targets and evolution of resistance mechanisms. Additionally, many aspects of apicoplast translation and its divergence from bacterial systems remain poorly understood. Addressing these knowledge gaps is essential for optimising malaria treatment strategies, making best use of safe, cheap drugs, and mitigating the spread of resistance.

## CRediT authorship contribution statement

**Jessica L. Home:** Writing – review & editing, Writing – original draft. **Geoffrey I. McFadden:** Writing – review & editing. **Christopher D. Goodman:** Writing – review & editing.

## Funding

This work was supported by a 10.13039/501100000925National Health and Medical Research Council (NHMRC) Project Grant to GMcF and CDG, plus a NHMRC Investigator Grant to GMcF.

## Conflict of interest

The authors declare no conflict of interest
